# Cost-effectiveness and feasibility of conditional economic incentives and motivational interviewing to improve HIV health outcomes of adolescents living with HIV in Anambra State, Nigeria

**DOI:** 10.1186/s12913-021-06718-4

**Published:** 2021-07-11

**Authors:** Obinna Ikechukwu Ekwunife, Chinelo Janefrances Ofomata, Charles Ebuka Okafor, Maureen Ugonwa Anetoh, Stephen Okorafor Kalu, Prince Udegbunam Ele, George Uchenna Eleje

**Affiliations:** 1 Department of Clinical Pharmacy and Pharmacy Management, Nnamdi Azikiwe University, Awka, Nigeria; 2 Centre for Applied Health Economics, School of Medicine and Dentistry, Griffith University, Brisbane, Queensland Australia; 3 Virology Laboratory, Nnamdi Azikiwe University Teaching Hospital, Nnewi, Nigeria; 4 Division of Respiratory Medicine, Department of Medicine, Nnamdi Azikiwe University Teaching Hospital, Nnewi, Nigeria; 5 Department of Obstetrics and Gynaecology, Nnamdi Azikiwe University Teaching Hospital, Nnewi, Nigeria

**Keywords:** Cost-effectiveness, Feasibility studies, Incremental cost-effectiveness ratio, HIV/AIDS, Adherence, Adolescents, In-depth interviews

## Abstract

**Background:**

In sub-Saharan Africa, there is increasing mortality and morbidity of adolescents due to poor linkage, retention in HIV care and adherence to antiretroviral therapy (ART). This is a result of limited adolescent-centred service delivery interventions. This cost-effectiveness and feasibility study were piggybacked on a cluster-randomized trial that assessed the impact of an adolescent-centred service delivery intervention. The service delivery intervention examined the impact of an incentive scheme consisting of conditional economic incentives and motivational interviewing on the health outcomes of adolescents living with HIV in Nigeria.

**Method:**

A cost-effectiveness analysis from the healthcare provider’s perspective was performed to assess the cost per additional patient achieving undetected viral load through the proposed intervention. The cost-effectiveness of the incentive scheme over routine care was estimated using the incremental cost-effectiveness ratio (ICER), expressed as cost/patient who achieved an undetectable viral load. We performed a univariate sensitivity analysis to examine the effect of key parameters on the ICER. An in-depth interview was conducted on the healthcare personnel in the intervention arm to explore the feasibility of implementing the service delivery intervention in HIV treatment hospitals in Nigeria.

**Result:**

The ICER of the Incentive Scheme intervention compared to routine care was US$1419 per additional patient with undetectable viral load. Going by the cost-effectiveness threshold of US$1137 per quality-adjusted life-years suggested by Woods et al., 2016, the intervention was not cost-effective. The sensitivity test showed that the intervention will be cost-effective if the frequency of CD4 count and viral load tests are reduced from quarterly to triannually. Healthcare professionals reported that patients’ acceptance of the intervention was very high.

**Conclusion:**

The conditional economic incentives and motivational interviewing was not cost-effective, but can become cost-effective if the frequency of HIV quality of life indicator tests are performed 1–3 times per annum. Patients’ acceptance of the intervention was very high. However, healthcare professionals believed that sustaining the intervention may be difficult unless factors such as government commitment and healthcare provider diligence are duly addressed.

**Trial registration:**

This trial is registered in the WHO International Clinical Trials Registry through the WHO International Registry Network (PACTR201806003040425).

**Supplementary Information:**

The online version contains supplementary material available at 10.1186/s12913-021-06718-4.

## Background

Adolescents living with HIV (ALHIV) have been tagged with worse treatment outcomes [1], with no evidence of practicable cost-effective interventions [2]. Previous studies showed that they had poor inclusion in care, poor knowledge of their HIV status, poor linkage to HIV care and poor retention in care compared to other age groups [2–4]. All these have led to very poor adherence to antiretroviral therapy, lower rates of viral load suppression and an increase in HIV-related morbidity and mortality in the adolescent population worldwide [2]. These outcomes are worse in sub-Saharan Africa where more than eight out of every ten of the world’s ALHIV live [2]. Adherence challenges identified include poverty, poor mental and physical health, the lack of a school system that is responsive to their needs, challenges in status disclosure, various forms of stigmatization, and challenges of medical adherence leading to the need for close monitoring [5].

Retention in HIV care is vital for achieving antiretroviral therapy (ART) adherence and viral suppression. Through clinical visits, patients in care are encouraged to initiate ART, refill their medications, medication side effects monitored, treatment failures diagnosed, and when necessary, switched to second- or third-line ART regimens. Retention-in-care, therefore, helps patients to maintain high medication adherence, thereby achieving viral suppression, improving health outcomes, reducing individual and population costs and reducing the risk of horizontal transmission.

Retaining patients in care over time is problematic especially for the highly vulnerable adolescents. Poor retention-in-care and adherence to ART in adolescents may be connected to the unique psychological, social and health needs that are peculiar to the adolescent stage [6]. These coupled with lack of interventions channelled to the care of adolescents [2] have led to a 30% increase in new HIV infections among adolescents and youth (15–24 years) as well as increased HIV-related mortality and morbidity in adolescents with HIV unlike in other age groups [3], making HIV the second leading cause of death among adolescents globally [6].

Despite the increasing number of HIV-focused service delivery interventions, adolescents are constantly left behind in HIV care services that would improve diagnosis, linkage to care, adherence to ART, and retention-in-care [7]. Since adolescence is a growth phase with specific healthcare and developmental needs, it is imperative to have interventions tailored to meet the unique needs of this population.

Various interventions to improve adolescents’ ART adherence and retention in care have been studied. MacPherson et al in 2015 carried out a systematic review to evaluate the effectiveness of service delivery interventions to improve adolescents’ linkage, retention and adherence to ART [1]. Eleven studies carried out in the last thirteen and a half years from the year of the study were reviewed, with the majority of them being conducted in high-income countries. Only 3 studies were conducted in countries with a high burden of the HIV epidemic and where there is a higher concentration of the world’s HIV-positive adolescents. Service delivery interventions identified were peer support, motivational interviewing, counselling, education, directly observed treatment and financial incentives.

The combination of conditional economic incentives (CEI) and motivational interviewing are theoretically promising interventions to improve HIV health outcomes in adolescents. While CEI produces an immediate improvement, motivational interviewing brings about long-lasting change as good behaviour is internalized [6]. However, in a resource-limited setting, it is important to determine whether allocating resources to these interventions would yield good value for money. Cost-effectiveness analysis (CEA) is the most common health economic evaluation tool used to compare at least two interventions, to identify the intervention that has a higher likelihood of producing the greatest health gains with the least resources [8]. Incremental cost-effectiveness ratio (ICER) is used to compare and rank alternative interventions..

## Study aim

This study was based on a cluster randomized controlled trial and aimed to assess the cost-effectiveness and feasibility of conditional economic incentives coupled with individualized face-to-face motivational interviewing on HIV health outcomes (including undetected viral load, retention-in-HIV care and adherence to antiretroviral therapy) of adolescents living with HIV in Anambra State, Nigeria.

## Methods

### Description of the cluster-randomized trial

This research was piggybacked on a cluster randomized controlled trial (RCT). The trial was registered in the WHO International Clinical Trials Registry through the WHO International Registry Network on 02/02/2018 (PACTR201806003040425). Ethical permission for the cluster-randomized trial was gotten from Nnamdi Azikiwe University Teaching Hospital Ethics Committee (NAUTH/CS/66/VOL11/092/2018/052). Twelve hospitals were selected from HIV treatment hospitals in Anambra state that were registered by the National Agency for the Control of AIDS (NACA) to render such services and were regarded as the clusters. The hospitals were then paired as a unit according to the type of hospital (e.g. secondary or tertiary). The units were randomly allocated to either the intervention or control group, such that each group had six hospitals. The trial had two periods (intervention and post-intervention) and each period had a duration of a year. The intervention was applied to the intervention group for 1 year (intervention period) while the control group received routine HIV-care. The intervention group received US$5.6 when the viral load is < 20 copies/ml for the first 3 months, received US$2.8 if the viral load remained suppressed for the next 3 months and the next 6 months, and then received US$5.6 if the viral load remained < 20 copies/ml for the next 1 year. This cash reward was linked to attaining undetected viral load and also attending motivational interview sessions on each clinic visit. Further information on the methods can be found on the trial protocol [6], while the completed trial is currently under peer review for publication (unpublished data).

The primary outcome was the difference in the proportion of participants with an undetected viral load between the two groups (< 20 copies/ml) at the twelfth month. The secondary outcomes were the adherence to ART and hospital appointment, CD4+ count outcome and retention in care.

### Cost-effectiveness analysis

Costing was based on a health provider’s perspective and the base year for the analysis was 2019. The type, quantity and unit prices of all resources used during the 1 year trial examining the impact of the incentive scheme on undetected viral load were documented prospectively for the intervention arm and control arm. Financial accounts of the hospitals used for the study and patient case folders were also reviewed where necessary. The costs of the research exercise such as the cost of data collection by the study team were excluded. To determine the cost for each resource used in the trial, we multiplied the quantity of resource consumed by unit cost. The total cost was derived by adding up all the individual costs. All costs were expressed in 2019 United States Dollars (USD) at an exchange rate of NGN360 per USD.

The personnel cost per HIV client in all the 12 hospitals was calculated based on the annual salaries as provided by the healthcare personnel. The average cost of motivational interviewing was then obtained by calculating the proportion of the average annual salary that accounts for the personnel’s time invested in offering motivational interviewing during the 1 year trial period.

Antiretroviral (ART) therapy used by patients were collected during the trial from resource use data and their costs estimated using prices obtained from the Global Fund’s Pooled Procurement Mechanism Reference Pricing for ART [9]. The estimation of costs of additional non-ART was based on the buyer’s median price of the International Medical Products Price Guide [10]. This was adjusted to 2019 prices using the gross domestic product (GDP) deflators.

The average cost of laboratory test (viral load) was obtained from the Virology Laboratory of Nnamdi Azikiwe University Teaching Hospital while that of CD4 count was obtained from the CD4 laboratories of St. Charles Borromeo Hospital Onitsha, St Joseph Adazi-Nnukwu and Nnamdi Azikiwe University Teaching Hospital where the tests were carried out. The cost per outpatient visit at the health centres was obtained from a survey of HIV clinics in Nigeria [11], which was also adjusted to 2019 values. The inpatient costs were derived from estimations of the cost of the basic healthcare package for hospitalized patients, including additional laboratory tests. Other recurrent costs obtained were the cost of transportation of samples to the designated laboratories for investigation and the cost of phone calls to track patients. Details of the cost analysis are shown in supplementary file 1.

A cost-effectiveness analysis was conducted to assess the cost per additional patient achieving viral suppression through the proposed intervention. The cost-effectiveness of the incentive scheme and motivational interviewing over routine care was estimated using the ICER, expressed as cost/patient who achieved an undetectable viral load [12].

$$ ICER=\frac{Cost\kern0.17em of\kern0.17em Intervention\hbox{-} Cost\kern0.17em of\kern0.17em Routine\kern0.17em care}{Effectiveness\kern0.17em of\kern0.17em Intervention\hbox{-} Effectiveness\kern0.17em of\kern0.17em Routine\kern0.17em care} $$

The cost-effectiveness threshold of US$1137 (i.e., 0.51 times the GDP of Nigeria in 2019) per quality adjusted life year (QALY) suggested by the Woods et al., 2016 was used to judge whether the intervention was cost-effective [13]. Although our study did not measure benefit in QALY, we used this cost-effectiveness threshold as a proxy to guage the cost-effectiveness of the Incentive Scheme intervention assuming that achieving undetected viral load will eventually lead to one living a normal life, rather than dying from HIV/AIDS. .

### Assessment of feasibility

Consent was obtained from all subjects in the trial. A qualitative study using an in-depth interview (IDI) was conducted on the healthcare personnel in the intervention arm, who administered the incentive scheme in the trial (three from each hospital/cluster). The IDI guide used was developed using the Pathfinder International Tool Series guideline on conducting IDI [14]. The IDI was a phone interview during which notes, and audio digital recording was taken. All recorded data were independently transcribed verbatim into the English language. A thematic content approach, guided by the Graneheim and Lundman framework, was utilized for analyzing the qualitative data [15]. Responses from the IDIs were read through systematically and repeatedly to get familiar with them, as well as to identify the meaning units. A meaning unit was identified as a string of the text that expressed a single coherent thought, up to the point where the coherent thought changed. The meaning units correspond to different codes which describes the idea or feeling expressed in the meaning units. Codes concerning the same subject were grouped into categories called themes. Information obtained during the IDIs was analyzed and merged according to the codes, themes and sub-themes. The original data were also reassessed after analysis to detect any idea or information that may have been missed.

### Statistical analysis

Cost analysis was conducted using Microsoft Excel (version 2007). Cost data were expressed as mean. We performed a univariate sensitivity analysis to examine the effect of key parameters effect on the ICER. We varied the major cost estimates by +/− 25% in the sensitivity analysis. We also considered a reduction in the frequency of CD4 and viral load tests in the intervention arm. Given that the trial occurred during the period that HIV patients were transitioned to Dolutegravir-based combination, we also assessed the effect of the regimen change to Dolutegravir-based combination on the ICER .

## Results

### Cost-effectiveness analysis

The effectiveness of the intervention is shown in Table 1. The difference in the percentage of participants with an undetected viral load between the participants in the intervention group and those in the control group was 11.7%. However, the difference in the percentage of participants with an undetected viral load between the participants in the intervention group and those in the control group reduced to 8.9% when adjusted for study participants with regimen change to Dolutegravir-based combination.

**Table 1:** Effectiveness of the intervention on the proportion of subjects with undetected viral load *(≤20 copies/ml)* (*N* = 246)

	Intervention		Control		*P*-value
	Number with undetected viral load (%)	Change over 12 months (E_12_–E_0_)	Number with undetected viral load (%)	Change over 12 months (E_12_ – E_0_)	
At baseline	26/119 (21.8%)	–	54/127 (42.5%)	–	< 0.001
At 12 months	38/119 (31.9%)	10.1%	52/127 (40.9%)	−1.6%	0.15
At 12 months adjusted^a^	28/77 (36.4%)	14.6%	27/56 (48.2%)	5.7%	0.12

^a^Adjusted for study participants with regimen change to Dolutegravir-based combination

The intervention cost a total of US$338.70 per patient/year while routine care cost a total of US$172.68 per patient/year. The main cost component in the intervention arm was the viral load tests as the participants in the intervention arm had four viral load tests per year compared to the participants in the control arm that had one viral load test per year. Other cost drivers include the ART, non-ART, and the CD4 count tests (which was also performed 4 times in the intervention arm compared to one time in control arm). Further details of the cost analysis are shown in Table 2.

**Table 2:** Average cost per patient in each cohort

Cost item	Average cost (USD)	Data source
***Intervention***		
Outpatient cost	8.31	[11]
Inpatient cost	0.9	Health facilities
Motivational interviewing	0.0032	Health facilities
Economic incentives	4.78^a^	Health facilities
CD4 Count test	22.4	Health facilities
Viral Load test	133.3	Health facilities
Transportation of samples to lab	22.4	Health facilities
Medication costs	139.51	[10]
Phone calls to track patients	0.19	Health facilities
Additional laboratory investigations	6.91	Health facilities
***Total***	*338.70*	
***Control arm***		
Outpatient cost	8.31	[11]
Inpatient cost	0.00	Health facilities
CD4 Count test	5.6	Health facilities
Viral Load test	33.3	Health facilities
Transportation of samples to lab	5.6	Health facilities
Medication costs	117.87	[10]
Phone calls to track patients	0.16	Health facilities
Additional laboratory investigations	1.84	Health facilities
***Total***	*172.68*	

^a^ For patients who received the incentive, the average total amount received was US$16.84

The ICER of the intervention compared to the routine care was US$1419 per additional patient with undetected viral load. A sensitivity test by adjusting the effectiveness outcome for regimen change to Dolutegravir-based combination increased the ICER to US$1865 per additional patient with undetected viral load. Varying the cost estimates of the cost drivers by +/− 25% changed the ICER to values between US$1169 and US$1669 per additional patient with undetected viral load. However, if the CD4 count and viral load tests are performed triannually, the ICER will become US$1086 per additional patient with undetected viral load, which is cost-effective (Table 3).

**Table 3:** Incremental cost-effectiveness ratio of the intervention and the sensitivity analysis

		Cost of HIV care in the intervention arm (USD)	Cost of HIV care in the control arm (USD)	Cost difference (USD)	Effectiveness (%)	ICER ($US/ additional patient with undetectable viral load)
	Main model	338.70	172.68	166.02	0.117	1419
Sensitivity analysis	Dolutegravir-based combination	338.70	172.68	166.02	0.089	1865
	Changes in the cost of ART (±25% [L, U])	317.00 & 360.4	145.35 & 200.00	171.65 & 160.40	0.117	1467 & 1371
	Changes in the cost of non-ART (±25% [L, U])	325.52 & 351.88	170.54 & 174.82	154.98 & 177.06	0.117	1325 & 1513
	Changes in the CD4 and viral load tests costs (±25% [L, U])	299.78 & 377.63	162.95 & 182.41	136.83 & 195.22	0.117	1169 & 1669
	Triannual CD4 and viral load tests for the intervention arm	299.80	172.68	127.12	0.117	1086
	Changes in viral load tests cost (±25% [L, U])	305.37 & 372.03	164.36 & 181.01	141.01 & 191.02	0.117	1205 & 1632
	Changes in the cost of ART, CD4, and viral load tests (±25% [L, U])	278.08 & 399.32	135.62 & 209.74	142.46 & 189.58	0.117	1218 & 1620

*[L, U]* lower and upper limit, *ICER* incremental cost-effectiveness ratio, *ART* antiretroviral therapy

### Feasibility studies

Out of eighteen healthcare professionals in the intervention arm of the study, fifteen of them (which included 4 Doctors, 4 Pharmacists, 1 Nurse and 6 Medical Laboratory Scientists) participated in the in-depth interview. The other three were on several attempts not available for the interview. The responses to the interview questions were classified using the thematic content analysis into major themes, including their sub-themes (Table 4).

**Table 4:** Themes and sub-themes from the interview

S/N	Theme	Sub-Theme	No of Persons Who Reported this
**1.**	Improved adherence	• The incentives encouraged them to adhere to treatment • The consequent reduction in viral load encouraged them the more • The adolescents became more open during motivational interviewing • Peer pressure from those who received the incentives encouraged others • The economic incentives spurred them to keep monthly appointments • Some of them used their incentives to supplement their transportation fares as this was a challenge	15
**2.**	Attitude towards the disease	• The adolescents and their parents/caregivers gained a better understanding of the disease condition • A few of them showed a negative attitude by being rebellious, especially those who still blamed their parents for infecting them	. 15
**3.**	Sustainability concerns	• Intervention implementation is very possible but would require a deep sense of commitment from the government, healthcare providers and other actors • Intervention may not be feasible in the long run • Too much workload on healthcare staff thus may require an increase in remuneration • Lack of political will from the government as other costs of HIV care are already being covered by the government	15
		• Economic incentives may not be available in a real-life setting, therefore other types of incentives may have to be considered for example skill acquisition programs for the adolescents. • Modification of the traditional adherence counselling to an intensified adherence counselling such as motivational interviewing. • May require capacity building for the motivational interviewers	
**4.**	Healthcare provider- adolescent relationship	• The motivational interviewing and regular visits brought about an improved relationship between the healthcare providers and the adolescents	10
**5.**	Caregivers’ influence	• Some adolescents depend on their caregivers who may not be disposed to bring them to the hospital for care and for laboratory investigations when needed. • Poor disclosure habit which inhibited the adolescents’ proper understanding of the disease condition.	15
**6.**	Cost implication	• Phone calls and short message service (SMS) to remind the adolescents of their monthly appointments/laboratory investigations • Use of phone consultations for those living far away.	13

Seven major themes and twenty-six sub-themes were identified. The identified themes were improved adherence, attitude towards the disease, sustainability concerns, healthcare provider-adolescent relationship, caregivers influence, cost implications, and intervention implementation challenges.

Some of the excerpts from the themes as stated by the healthcare professionals interviewed are shown below:

- **Improved adherence**: From the perspective of the healthcare professionals interviewed, the economic incentives and motivational interviewing played a huge role in improving ART adherence and reducing viral load.

Positive peer group influence was observed as an adherence-stimulating factor as those who didn’t receive the monetary benefits felt compelled to do so on seeing their peers rewarded. A medical laboratory scientist involved in the trial stated that:

*“The economic incentives spurred them to adhere to their treatments, especially those who had not been adhering. Out of 14 enrolled adolescents in our arm, 9 achieved viral load suppression. Those who didn’t achieve suppression felt remorse on seeing others get monetary benefits and were challenged to do better. The parents/caregivers of those who did well were very happy and more willing to encourage their children/wards”.* (Medical Laboratory Scientist, Community Health Centre, Neni)

- **Attitude towards the disease:** The intervention brought about a positive attitude in the participants. When the adolescents got enrolled in the study, they were made to understand what it was about. As they experienced a drop in their viral load, they understood the relationship between adherence to treatment and virological and immunological outcomes. They, therefore, became more optimistic about their health outcomes. A medical laboratory scientist involved in the trial stated that:

*“Before their enrollment into the study, some adolescents didn’t know what they were taking drugs for. But during the intervention implementation, we made them understand how the ART worked and how their health conditions would improve upon adhering to treatment. Hence, they became eager to take charge of their health.”(*Medical Laboratory Scientist, Community Health Centre, Neni).

Some participants in the intervention still displayed a negative attitude. Although there was high patient acceptability of the intervention, a few others who still blamed their parents for infecting them were indifferent. A doctor involved in the trial informed us that:

*“Some of the adolescents still exhibited a nonchalant attitude towards the incentive scheme. They still felt animosity towards their parents for infecting them and felt they would, therefore, be doing them a favour by taking their drugs religiously.” (*Doctor, Nnamdi Azikiwe University Teaching Hospital, Nnewi).

- **Healthcare personnel and HIV adolescents’ relationship:** The motivational interviewing fostered the relationship and allowed for better interaction between the healthcare providers and adolescents. A pharmacist involved in the trial implementation stated that:

*“The monthly motivational interviewing made the adolescents open up the more and enabled me to gain a better understanding of the challenges to adherence that they faced. Therefore, we were able to work together to address these challenges for a better outcome.”* (Pharmacist, Nnamdi Azikiwe University Teaching Hospital, Nnewi)

- **Sustainability of the intervention:** Most of the interviewees saw the intervention as worthwhile but expressed doubts about the possibility of sustaining the intervention in the long run unless there is a great sense of commitment on the part of both the government and the healthcare providers. A nurse involved in the trial implementation stated the following:

*“The government already has a lot on their plates as regards HIV/AIDS management. Some of the costs of care that were initially borne by HIV support agencies are now born by the HIV clients, Therefore, I don’t think it will be feasible for the government to sustain these economic incentives in the long run*”. (Nurse, St. Joseph’s Hospital Adazi-Nnukwu)

One of the interviewees, however, stated that instead of monetary rewards which may be stopped halfway, other forms of incentives which can help build entrepreneurial skills in these adolescents and thus a source of financial empowerment could be combined with motivational interviewing to achieve the intervention sustainability. He said:

“*Sustainability would require more than financial incentives. Skill acquisition programs could be designed for these adolescents as a way of empowering them to make some money to take care of their transportation fares to the hospital”.* (Doctor, Community Health Centre, Neni)

Another participant suggested that an increase in the healthcare personnel’ remuneration may boost their morale as it will be extra work added to their already loaded schedule. She said:

*We already have a lot of workloads here at the HIV unit, therefore additional remuneration could serve as a form of encouragement”.* (Pharmacist, General Hospital Onitsha)

- **Caregiver’s influence:** Because younger adolescents lacked the autonomy to get themselves to the hospitals for appointments or laboratory investigations, they had to wait on their caregivers who may be pre-occupied with other activities/engagements. A medical laboratory scientist involved in the trial stated that:

“*The adolescents didn’t present for laboratory tests as and when due. Most of them depended on their parents who may not be disposed to bring them to the hospital when they are supposed to.”(*Medical Laboratory Scientist, St. Charles Borromeo Hospital, Onitsha)

- **Cost implications:** Fear of stigmatization was found to influence parents’/caregivers’ choice of hospitals to enrol their children/wards in. They, therefore, preferred hospitals far away from their neighbourhood. This came with the challenge of the high cost of transportation, thus impacting negatively on their attendance at the monthly appointments. A doctor involved in the trial informed us that:

*“Some of the caregivers are afraid of being seen as HIV positive when they are not. Some who do not want their children/wards to be identified by friends or relatives as HIV client would rather enrol in hospitals far from their places of residence. This made it difficult for them to keep up with their monthly appointments due to the increased cost of transportation.”* (Doctor, Immaculate Heart Hospital and Maternity, Onitsha)

Also, the healthcare providers had to continuously use phone calls or text messaging to remind the adolescents and/or their parents/caregivers about monthly appointments/laboratory investigations.

- **Intervention implementation challenges:** A lot of challenges were experienced by the program implementers during the trial and they include low attendance for the quarterly laboratory investigations, distance barriers, death of some clients, failure of samples to show results and difficulties in tracing lost-to-care clients. A medical laboratory scientist stated that:

*“Some of the adolescents didn’t understand that the laboratory investigations benefitted them more than it even benefitted the investigators. They felt that they were being pressured to attend compulsory laboratory investigations.” (*Medical *Laboratory Scientist,* St. Joseph’s Hospital Adazi Nnukwu, Nigeria)

Some healthcare providers found it difficult to get those who were lost-to-care to initially enrol in the clinical trial and also for continuation in the study for those enrolled as their phone numbers remained unavailable.

Besides, some samples for viral load assay kept failing. Therefore, it was burdensome for the affected adolescents to continue coming for repeated laboratory investigations and yet not knowing his or her viral load status and eligibility for the financial incentive. A laboratory scientist involved in the trial stated that:

*A particular sample kept on failing on the test without a reason. The client felt reluctant to repeatedly present for more tests. An investigation is still on however to know the cause of that in a particular client in my facility. Only his baseline viral load result was successful.” (*Laboratory Scientist, Community Health Centre, Neni, Nigeria)

## Discussion

This study showed that the incremental cost-effectiveness ratio expressed as the incremental costs per patient with undetectable viral load was US$1419. Going by the cost-effectiveness threshold suggested by Woods et al., 2016 [13], the Incentive Scheme was not cost-effective. The sensitivity analyses showed that adjusting the effectiveness outcome for regimen change to Dolutegravir-based combination, and changes in the cost estimates of the cost drivers by +/− 25% did not yield ICER below the threshold. However, if the CD4 count and viral load test are performed at most triannually, the intervention will become cost-effective. Thus, the ICER obtained in our study showed that the intervention was not cost-effective, but will become cost-effective if the key laboratory health indicators (CD4 count and viral load tests) for HIV patients are performed 1–3 times per annum.

On the feasibility assessment, the healthcare providers reported that patients’ acceptance of the intervention was very high, as evident from the regular attendance of monthly appointments. We gathered that the views of the healthcare providers on the new intervention could be placed into seven major themes: improved adherence, attitude towards the disease, sustainability concerns, healthcare provider-adolescent relationship, caregivers influence, cost implications and intervention implementation challenges.

The findings from the cluster-randomized trial and the view of the healthcare providers suggest that the Incentive Scheme increased the virologic outcomes of adolescents living with HIV. Previous studies on interventions to improve adolescents’ adherence to therapy have shown that monetary rewards influence behaviours [16–18]. In a single-centre adherence intervention combining financial incentives (total expenditure £1350) with motivational interviewing for adolescents with perinatally- acquired HIV infection, there was improved virological outcomes [16]. For young people who may not fully comprehend the implication of having to live with the virus, with the consequent lifelong use of medications, low adherence to regimen would be very common. Therefore, to increase adherence rates, it may be necessary to reward them for doing what they ought to be doing anyway. In our trial, the monetary rewards attracted the adolescents to the scheme, while the motivational interviewing provided an avenue for them to connect with the healthcare providers. This connection brought about an intrinsic motivation that would sustain lifelong positive behaviour change. The client-centred counselling style of motivational interviews likely helped the patients move away from a state of indecision and towards motivation to making positive decisions.

The expectation of monetary rewards was an extrinsic motivator. Unfortunately, it is known that cash incentive may not be able to bring about lifelong positive behaviour change. A few post-intervention evaluations found that adherence rates diminish when interventions are withdrawn [17, 18]. Motivational interviewing was found to foster a better relationship between healthcare providers and adolescents. It has also been found effective in influencing health behaviours, improving adherence and changing harmful lifestyle among people with chronic diseases [19]. In an integrative review to examine the use of motivational interviewing to improve health outcomes in persons living with HIV, it was discovered that studies using motivational interviewing either alone or in conjunction with other service delivery interventions, recorded improved adherence, decreased depression, and decreased risky sexual behaviour [20]. The IDI participants also reported a positive relationship between motivational interviewing and treatment adherence. It also improved the adolescents’ understanding of their disease condition.

The interventions hold promising prospects, but doubts have been expressed by the IDI participants about sustainability. Although none of them expressed frustrations about any disruptions in clinic workflow during the intervention implementation emanating from the trial, there were concerns about the increase in workload and financial requirements for the incentives in a real-life setting. Therefore, a team of dedicated healthcare providers would be needed to drive the intervention in a real-life setting. Additional remuneration for the staff involved was also recommended. Healthcare staff providing the motivational interviewing needs to exude confidence in their capacity to conduct it. Thus, the capacity building of healthcare staff on motivational interviewing would enhance the impact of the intervention. The skillset required for successful implementation of motivational interviewing includes but not limited to gaining an understanding of the philosophy behind it, acquisition of basic client-centred counselling skills, as well as recognizing and reinforcing change [21].

Another genuine concern expressed by the health care workers is the financial sustainability of the Incentive Scheme. We believe that the Incentive Scheme can be sustained financially by incorporating it as one of the social welfare schemes that are operational in Nigeria such as the school feeding programme [22] and *Tradermoni* [23]. The Incentive Scheme could be targeted to assist adolescents who cannot afford transportation fares for hospital appointments or applied to hospitals in rural and poor settings as a way of reducing the cost of implementation. Incentive scheme as social welfare programme will have the dual purpose of improving the health and economic status of the beneficiaries. The healthcare providers indicated that the orphaned adolescents were cared for by guardians who may have limited earning capacity. This places immense financial stress on them as much of their income is spent on household expenses. Also, the interviewees stated that the majority of the HIV adolescents came from poor backgrounds and hence have difficulties affording transportation fares to the hospitals. A lot of HIV adolescents need financial support for self-sustenance especially as some of them have lost at least one parent to HIV/AIDS.

The study had some limitations. One major limitation of the study was the lack of sample size adequacy. Some of the hospitals had a low client load and could not recruit up to the expected sample size (23 adolescents living with HIV). Thus, the trial did not reach the desired sample size. Given that retention in care was one of the trial outcomes, we could not influence retention in the study beyond what is done routinely in the hospitals used for the trial. Another limitation was that the baseline viral suppression in the intervention group was significantly lower than in the control group and more participants in the intervention arm changed to a more efficacious therapy during the 1 year period. Thus, in addition to the Incentive Scheme intervention, these factors could have contributed to a larger change in viral load suppression observed in the intervention arm. Also, since the effectiveness outcome was not measured as QALY, the cost-effectiveness threshold was used approximately to gauge the cost-effectiveness of the intervention. The interview was not all-inclusive as it focused on the healthcare personnel without also exploring the perspectives of the HIV clients who are the direct beneficiaries of the intervention. Therefore, we may have missed some vital information that may enhance or hinder the feasibility of the incentive scheme from the ALHIV. However, the interviewees bared their minds based on their several contacts with the adolescents. Also, the assessment of the effectiveness outcome was on a short-term basis. It was not clear whether the intervention effect could be sustained after intervention withdrawal, in which case the ICER would be lower, translating to a more efficient intervention.

Given the dwindling funding for HIV program, there is a need for careful consideration of any intervention to be included as part of the HIV program. In this study, we have illustrated how cost-effectiveness analysis can be applied to an HIV service delivery intervention to establish its efficiency. Such analysis will better enable public health decision-makers to determine health interventions that will produce the greatest benefits given resource constraint.

## Conclusion

Our results showed that the Incentive Scheme comprising of conditional economic incentives and motivational interviewing was not cost-effective based on our ICER threshold. However, it will become cost-effective if the frequency of the CD4 count and viral load tests for the intervention arm are reduced to 1–3 times per annum. The acceptance of the intervention was very high from the perspective of health professionals, but the health professionals believed that sustaining the intervention may be difficult unless factors such as government commitment and healthcare provider diligence are duly addressed.

## Supplementary Information

**Additional file 1.**

## Data Availability

The datasets used and/or analysed during this study are available from the corresponding author on reasonable request.

## Abbreviations

ART: Antiretroviral Therapy
HIV: Human Immunodeficiency Virus
ICER: Incremental Cost-effectiveness Ratio
IDI: In-depth-interview
WHO: World Health Organisation
NGN: Nigerian Naira
WHO-CHOICE: World Health Organization’s Choosing Inervention that is Cost Effective
GDP: Gross Domestic Product
AIDS: Acquired Immunodeficiency Syndrome
ALHIV: Adolescents Living with HIV
CEI: Conditional Economic Incentives
CEA: Cost-effectiveness Analysis
NACA: National Association for the Control of AIDS
NAUTH: Nnamdi Azikiwe University Teaching Hospital
HIV-RNA: Human Immunodeficiency Virus-Ribonucleic Acid
QALY: Quality Adjusted Life Years
UNAIDS: Joint United Nations Programme on HIV/AIDS
US: United States
