# Supplementary material for: Cost-effectiveness and feasibility of conditional economic incentives and motivational interviewing to improve HIV health outcomes of adolescents living with HIV in Anambra State, Nigeria
Source: BMC Health Serv Res. 2021 Jul 11;21:685. doi: 10.1186/s12913-021-06718-4 (PMC8272893; doi:10.1186/s12913-021-06718-4)
Supplement: Supplementary file 1 — Additional file 1. [file 12913_2021_6718_MOESM1_ESM.docx]

# Supplementary 1: Cost analysis of the ARA Trial

Number of participants in the intervention arm = 119 Number of participants in the control arm = 127

1. **Cost of antiretroviral therapy (ART), adjusted to 2019 USD values**

Table 1: Cost of ARVs for the control and intervention groups.

|  | Number of patients in the intervention arm (percentage) | Cost (USD) | Number of patients in the control arm (percentage) | Cost (USD) |
| --- | --- | --- | --- | --- |
| ZLN | 67 (56.3%) | 3276.3 | 45 (35.4%) | 2200.5 |
| TLE | 32 (26.9%) | 2208 | 43 (33.9%) | 2967 |
| TLD | 5 (4.2%) | 333 | 16 (12.6%) | 1065 |
| ABC/3TC + LPV/r | 2 (1.7%) | 463.3 | 8 (6.3%) | 1853.2 |
| ZDV/3TC+LPV/r | 2 (1.7%) | 990 | 7 (5.5%) | 3465 |
| TDF/3TC+LPV/r | 5 (4.2%) | 2364 | 4 (3.1%) | 1891.21 |
| ABC/3TC/EFV | 5 (4.2%) | 642 | 3 (2.4%) | 385.2 |
| ZLE | 1(0.8%) | 49.1 | 1(0.8%) | 49.1 |
| Total cost (USD) |  | 10326.1 |  | 13876.6 |
| Cost of ART per patient (Intervention arm) =10326.1/119 = 86.8USD  Cost of ART per patient (Control arm) = 13876.6/127 = 109.3USD | | | | |

1. **Cost of non-antiretroviral therapy (ART), adjusted to 2019 USD values**

Table 2: Cost of non-ART for the control and intervention groups.

|  | Number of patients | Medication cost (USD) | Cost per patient (USD) |
| --- | --- | --- | --- |
| Intervention arm | 119 | 6273 | 52.71 |
| Control arm | 127 | 1088 | 8.57 |

**Note:** The non-ART included antimalarials, anti-fungals, antibacterials, antihelminthics, antivirals, anti-tussives, antipyretics, analgesics, immune boosters, antidiarrhoeals, Vitamin supplements, anti-ulcers, drugs for tuberculosis prophylaxis, blood tonics and blood for transfusion.

1. **Cost of healthcare staff involved in direct care of the patients at the HIV unit**

Table 3: Cost of staff involved in direct patient care in the HIV clinics

| **Hospital** | **Health staff cadre** | **Annual salary**  **(USD)** |
| --- | --- | --- |
| Nnamdi Azikiwe University Teaching Hospital | Doctor | 6699 |
|  | Pharmacist | 5159 |
|  | Medical Laboratory Scientist(MLS) | 4726 |
| Community Health Centre | Doctor | 5588 |
|  | Pharmacist | 5159 |
|  | MLS | 5282 |
| St Joseph’s Hospital | Doctor | 4000 |
|  | Nurse | 1167 |
|  | MLS | 2167 |
| General Hospital | Doctor | 4000 |
|  | Pharmacist | 3100 |
|  | MLS | 3000 |
| St. Charles Borromeo | Doctor | 3944 |
|  | Pharmacist | 2467 |
|  | MLS | 2167 |
| Immaculate Heart Hospital and Maternity | Doctor | 4722 |
|  | Nurse | 1167 |
|  | MLS | 2167 |
| Total cost of healthcare staff involved in the direct care of the patients = USD 66,681  Total number of all HIV clients (including ARA clients) in the 12 study sites = 31,168  Cost of care per patient=USD 66681/31,168 = USD 2.14 | | |

1. **Cost of motivational interview**

Time spent per motivational interview (MI) per patient=14.7 mins

Staff work: 5 days (per week) x 52 weeks = 260 days/year

Public holidays in the year 2019= 12days

Work days: 260 – 12 = 248 days

Each work day has 60mins x 8 hrs = 480 mins/day

Therefore each staff works for 480mins x 248 days = 119,040 mins in a year.

Total time for MI per patient per annum = 14.7 mins x 12 times in a year = 176.4 mins in a year.

Proportion of staff time used to interview a patient per annum = 176.4/119,040 * 100 = 0.148%

Cost of health care staff per patient= USD 2.14

Cost of MI per patient = 0.00148*2.14= USD 0.0032 per patient.

Table 4: Summary of cost calculations for the motivational interview (MI)

| Work days per year  (excluding public holidays) | Work time per day (mins) | Work time per year (mins) | Average monthly MI time (mins) | Annual MI time (mins) | Cost of health care staff per patient (USD) | Proportion of staff time for MI per year (%) | Yearly cost of MI (USD) |
| --- | --- | --- | --- | --- | --- | --- | --- |
| 248 | 480 | 119,040 | 14.7 | 176.4 | 2.14 | 0.148 | 0.0032 |

1. **Average annual cost of the recurrent expenditure**:

These include viral load test, CD4 count test, sample transportation and follow-up phone calls for the intervention and control groups.

Table 5: Average cost of viral load and CD4 tests, sample transportation, and follow-up phone calls for the intervention and control arm

|  | **Intervention arm** | | | **Control arm** | | |
| --- | --- | --- | --- | --- | --- | --- |
|  | Unit cost (USD) | Number of times per year | Cost per patient (USD) | Unit cost (USD) | Number of times per year | Cost per patient (USD) |
| Viral load test | 33.3 | 4 | 133.3 | 33.3 | 1 | 33.3 |
| CD4 count | 5.6 | 4 | 22.4 | 5.6 | 1 | 5.6 |
| Sample transportation | 5.6 | 4 | 22.4 | 5.6 | 1 | 5.6 |
| Follow-up by phone calls | -- | -- | 0.19 | -- | -- | 0.16 |
| Total cost per patient (USD) | 178.29 | | | 44.66 | | |

1. **Additional laboratory tests**

Table 6a: Cost of additional laboratory tests for the intervention arm

| **Type of test** | **Quantity** | **Unit cost (USD)** | **Total cost (USD)** |
| --- | --- | --- | --- |
| Malaria | 27 | 5.6 | 151.2 |
| Full blood count | 23 | 5.6 | 128.8 |
| Widal | 10 | 6.7 | 67 |
| Erythrocyte sedimentation rate | 8 | 1.8 | 14.4 |
| X-ray | 15 | 11.1 | 166.5 |
| Genexpert test | 80 | NGO-sponsored | Not applicable |
| Serum Urea and Creatinine | 23 | 11.1 | 255.3 |
| Urinalysis | 23 | 1.7 | 39.1 |
| Total cost = USD 822.3  Cost per patient in the intervention arm = 822.3/119 = USD 6.91 | | | |

Table 6b: Cost of additional laboratory tests for the control arm

| **Type of test** | **Quantity** | **Unit cost (USD)** | **Total cost (USD)** |
| --- | --- | --- | --- |
| Malaria | 1 | 5.6 | 5.6 |
| Widal | 1 | 6.7 | 6.7 |
| X-ray | 20 | 11.1 | 222 |
| Acid fast bacilli | 20 | NGO-sponsored | NA |
| Mantoux skin test | 20 | NGO-sponsored | NA |
| Total= USD 234.3  Cost per patient = 234.3/127 = USD 1.84 | | | |

1. **Cost of conditional financial incentives**

Table 7: The amount of incentives received at viral load evaluation times

| Time interval (months) | Number of patients | Unit amount of incentives given (USD) | Total amount of incentive (USD) |
| --- | --- | --- | --- |
| 3 | 33 | 5.6 | 184.8 |
| 6 | 35 | 2.8 | 98 |
| 9 | 32 | 2.8 | 89.6 |
| 12 | 35 | 5.6 | 196 |
| Total cost (USD | | | 568.4 |
| Average cost of conditional incentive for patients who achieved target viral load = 568.4/33.75 = USD16.84  Average cost per patient in the intervention arm = 568.4/119 = USD 4.78 | | | |

1. **Outpatient cost**

Outpatient cost based on Aliyu *et al,*2012 ^1^ = USD 7.4

Adjusted to 2019 value based on 2019/2012 GDP deflator ^2^ = 1.123*7.4 = USD 8.31

1. **Summary of cost estimates**

Table 8: Summary of the cost estimates

| **Items** | **Intervention arm (USD)** | **Control arm (USD)** |
| --- | --- | --- |
| Anti-retroviral therapy | 86.80 | 109.30 |
| Non-antiretroviral therapy | 52.71 | 8.57 |
| Motivational interview | 0.0032 | n/a |
| Recurrent expenditure (CD4 count test, viral load test, sample transportation, and phone call follow-up) | 178.29 | 44.66 |
| Additonal laboratory tests | 6.91 | 1.84 |
| Conditional incentive | 4.78 | n/a |
| Outpatient visit | 8.31 | 8.31 |
| Inpatient stay | 0.9 | n/a |
| **Total cost (USD)** | **338.70** | **172.68** |

References

1 Aliyu HB, Chuku NN, Kola-Jebutu A, Abubakar Z, Torpey K, Chabikuli ON. What is the cost of providing outpatient HIV counseling and testing and antiretroviral therapy services in selected public health facilities in Nigeria? *J Acquir Immune Defic Syndr* 2012; **61**: 221–5.

2 WorldBank. Indicators: Economy & Growth. 2019. https://data.worldbank.org/indicator/ (accessed Sept 10, 2020).
